# A transcriptomic and proteomic map of primary human cell types

**DOI:** 10.1093/nar/gkaf1498

**Published:** 2026-01-14

**Authors:** Dong-Gi Mun, Anil K Madugundu, Santosh Renuse, Raja Sekhar Nirujogi, Chan Hyun Na, Min-Sik Kim, Mayank Saraswat, Smrita Singh, Madan G Ramarajan, Shivani Tiwary, Jürgen Cox, Amol Prakash, Marc K Halushka, Kathleen H Burns, Richard K Kandasamy, Akhilesh Pandey

**Affiliations:** Department of Laboratory Medicine and Pathology, Mayo Clinic, Rochester, MN 55905, United States; Department of Laboratory Medicine and Pathology, Mayo Clinic, Rochester, MN 55905, United States; Institute of Bioinformatics, International Technology Park, Bangalore, Karnataka 560066, India; Manipal Academy of Higher Education, Manipal, Karnataka 576104, India; Department of Laboratory Medicine and Pathology, Mayo Clinic, Rochester, MN 55905, United States; Medical Research Council Protein Phosphorylation and Ubiquitylation Unit, School of Life Sciences, University of Dundee, Dundee DD1 5EH, United Kingdom; Department of Neurology, School of Medicine, Johns Hopkins University, Baltimore, MD 21205, United States; Institute for Cell Engineering, School of Medicine, Johns Hopkins University, Baltimore, MD 21205, United States; Department of New Biology, DGIST, Daegu 42988, Republic of Korea; New Biology Research Center, DGIST, Daegu 42988, Republic of Korea; Department of Laboratory Medicine and Pathology, Mayo Clinic, Rochester, MN 55905, United States; Department of Laboratory Medicine and Pathology, Mayo Clinic, Rochester, MN 55905, United States; Institute of Bioinformatics, International Technology Park, Bangalore, Karnataka 560066, India; Manipal Academy of Higher Education, Manipal, Karnataka 576104, India; Department of Laboratory Medicine and Pathology, Mayo Clinic, Rochester, MN 55905, United States; Institute of Bioinformatics, International Technology Park, Bangalore, Karnataka 560066, India; Manipal Academy of Higher Education, Manipal, Karnataka 576104, India; Computational Systems Biochemistry Research Group, Max Planck Institute of Biochemistry, Martinsried 82152, Germany; Computational Systems Biochemistry Research Group, Max Planck Institute of Biochemistry, Martinsried 82152, Germany; Optys Tech Corporation, Shrewsbury, MA 01545, United States; Pathology and Laboratory Medicine Institute, Cleveland Clinic Foundation, Cleveland, OH 44195, United States; Department of Oncologic Pathology, Dana-Farber Cancer Institute, Boston, MA 02115, United States; Department of Laboratory Medicine and Pathology, Mayo Clinic, Rochester, MN 55905, United States; Division of Computational Biology, Department of Quantitative Health Sciences, Mayo Clinic, Rochester, MN 55905, United States; Department of Immunology, Mayo Clinic, Rochester, MN 55905, United States; Department of Laboratory Medicine and Pathology, Mayo Clinic, Rochester, MN 55905, United States; Manipal Academy of Higher Education, Manipal, Karnataka 576104, India; Center for Individualized Medicine, Mayo Clinic, Rochester, MN 55905, United States

## Abstract

Molecular profiling of human primary cell types is essential for understanding human biology. We present a transcriptome and proteome map of 28 primary human cell types. Three major clusters of epithelial, endothelial, and mesenchymal cell types were observed in both the transcriptome and proteome levels along with the discovery of cell type enriched molecules including GRAP and C1orf116. The epithelial cell specific protein C1orf116 was further validated using immunohistochemistry across various human tissues. An exhaustive protein database search considering 39 post-translational modifications (PTMs) revealed novel insights into the PTM landscape including identification of understudied PTMs such as serine O-acetylation and histidine methylation. This also enabled comprehensive characterization of proteins with diverse PTMs. Interestingly, an unexpectedly higher frequency of dioxidation on tryptophan compared to methionine led to the identification of oxidative mitochondria complex subunit proteins. Further, a search strategy accounting for alternative translational start sites, splice junctions and translational readthrough refined genome annotation using proteomic evidence. For example, peptides from translational readthrough including extended sequence of LDHB and MDH1 were detected representing the first peptide-level evidence of these protein readthrough isoforms. Our comprehensive transcriptome and proteome data revealed cell type-specific molecular cues and heterogeneity, offering new insights into disease mechanisms often overlooked by tissue proteomics.

## Graphical Abstract

**Graphical Abstract:**
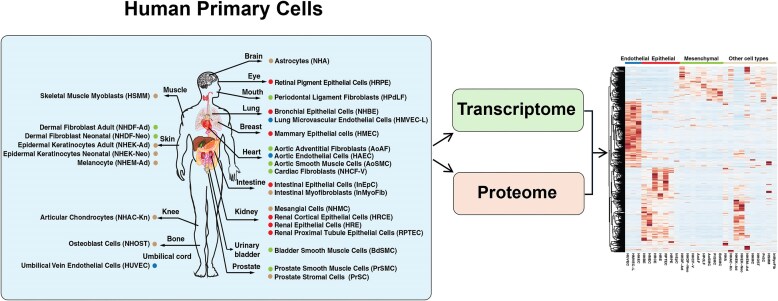

## Introduction

Understanding human biology is driven by the intricate analysis of molecular profiles of tissues and composite cell types under both normal and pathological conditions. Recent advancements in high-throughput sequencing and high-resolution mass spectrometry technology coupled with the development of new experimental and computational methods have enabled global analysis of biomolecules across a large number of samples. The Genotype-Tissue Expression (GTEx), Functional Annotation of the Mammalian Genome (FANTOM), and Encyclopedia of DNA Elements (ENCODE) consortia have produced invaluable resources of large-scale gene expression and associated gene regulatory information from multiple tissues and developmental stages of human [1–3]. The Human Protein Atlas (HPA) project provided a tissue-based map of the human proteome through transcriptomics and tissue microarray-based immunohistochemistry analysis [4, 5]. However, it is limited by its reliance on antibody-driven detection. Alternatively, mass spectrometry-based quantitative proteomics provided opportunities for comprehensive proteome profiling from cell lines and tissues [6]. We and others have previously presented the first proteome drafts of human tissues using high resolution mass spectrometry, which have been used to explore protein expression [7, 8]. Further efforts have been made to obtain comprehensive transcriptome and proteome data from 29 human normal tissues [9] and 201 samples having matched transcriptome data from GTEx [10]. These studies revealed proteins with tissue-enriched/specific expression and evaluated a correlation between messenger RNA (mRNA) and protein abundance. Although these efforts have greatly advanced our understanding of molecular cellular biology, the measurements have been focused on tissues consisting of millions of cells, generating average expression data for cells. Thus, a major shortcoming of these studies is the missed opportunity to assess cellular heterogeneity. Additionally, with the emergence of single cell sequencing technology, the Human Cell Atlas consortium has spearheaded international efforts to develop a detailed map of human cells [11, 12]. Database such as the Human Transcriptome Cell Atlas containing 19 adult and fetal tissues provide diverse transcriptomic profiles of cell types across tissues [13].

Primary cell cultures are a relevant model system for studying biological systems, as they are homogeneous and represent near physiological conditions, making them widely leveraged to understand cellular biology. Although several studies have analyzed transcriptome of primary cell types [14, 15], deeper profiling of genes and proteins in the context of human primary cell types has been less investigated. Therefore, we sought to generate the molecular profile of 28 different primary human cell types using RNA-seq and high-resolution mass spectrometry-based proteomics. We present a cell type map of the transcriptome and proteome of human primary cells encompassing the expression of mRNA, long non-coding RNAs (lncRNA), protein, a catalog of modified peptides, novel findings of missing proteins, alternative translational initiation sites, translation readthroughs, and splice variants. We anticipate that both transcriptome and proteome resources of primary human cell types will propel further fundamental and translational research.

## Materials and methods

### Cell culture

Primary human cells were acquired from Lonza and cultured following the manufacturer’s specifications. Briefly, 5 × 10^5^ cells were seeded on a 10-cm cell culture dish in culture medium supplemented with the essential growth factors and 2% fetal bovine serum. The cells were incubated in 5% CO_2_ at 37°C for six passages prior to the isolation of protein and RNA. The details of the culture conditions for each cells are summarized in Supplementary Table S1.

### Generation of RNA-seq data

Total RNA was isolated using RNeasy mini kit (Qiagen) following the manufacturer’s instructions. The quantity and integrity of the isolated RNA was assessed using Agilent BioAnalyzer. High‐quality DNase treated total RNA was used for library preparation using TruSeq stranded mRNA library preparation kit from Illumina and sequenced on HiSeq2500 according to the manufacturer’s guidelines. Briefly, mRNA was enriched from total RNA using poly(A) capture with oligo-DT beads followed by fragmentation and complementary DNA (cDNA) synthesis. The cDNA fragments were adenylated, adapter-ligated, and polymerase chain reaction-amplified to generate sequencing libraries, which were quality-checked using a BioAnalyzer and sequenced on an Illumina HiSeq 2500 platform (100 bp paired-end reads) as described previously [16]. Raw FASTQ sequence files were processed through Mayo’s internal MAp-RSeq pipeline (version 3.0) [17]. MAp-RSeq uses a variety of publically available bioinformatics tools tailored by in-house developed methods. In specific, the aligning and mapping of reads are performed via Star aligner against hg38 reference genome [18]. The gene and exon counts are generated by FeatureCounts [19] using the gene definitions files from GENCODE v33 and FANTOM v5. Quality control was carried out using RSeQC (version 4.0.0) [20] to ensure that the results from all samples are reliable and can be collectively used for downstream analysis.

### LncRNA analysis

Raw FASTQ sequence files were processed through Mayo’s internal UClncRNA pipeline (version 1.0.9) [21]. Specifically, transcript assembly was performed using TACO (version 0.7.3) [22] based on GENCODE v33 and FANTOM v5. Coding potential for novel lncRNA was assessed based on iSeeRNA [23] and CPAT [24]. Quantification was performed using FeatureCounts [19].

### Differential expression analysis to identify cell type and tissue-enriched genes

Transcript assembly and novel transcript quantification were performed using StringTie version 1.3.3 [25] and Differential exon usage analysis was performed using DEXseq version 1.32.0 [26]. Differential expression analysis was performed using R (version 3.5.2) with scripts developed utilizing the DESeq2 package. Genes with expression (counts per million) <0.01 were removed from the following differential analysis to reduce noises. *P*-value was adjusted for multiple hypothesis testing to control the false discovery rate (FDR) using Benjamini–Hochberg method. Differential expressed genes were selected based on adjusted *P*-values <.01 and absolute log_2_ fold changes >1. Pathway enrichment analysis was performed using enrichR v2.1.

### Protein extraction, digestion and fractionation

Cells were lysed in a lysis buffer (8 M GuHCl, 1 mM sodium orthovanadate, 2.5 mM sodium pyrophosphate, 1 mM β-glycerophosphate in 100 mM HEPES). After measuring protein concentration using a bicinchoninic acid (BCA) assay, about 200 µg of proteins were reduced and alkylated with 2.5 mM tris(2-carboxyethyl) phosphine, 5 mM chloroacetamide at room temperature for 30 min followed by precipitation using chilled acetone. The precipitated proteins were washed with methanol, and the remaining methanol was dried under vacuum. For digestion, 10 µg of sequencing grade trypsin in 500 µl of 100 mM triethylammonium bicarbonate was added and incubated at 37°C overnight. The peptides were dried and reconstituted in 200 µl of 1% trifluoroacetic acid and fractionated into six fractions using StageTip packed with strong cation exchange chromatography disks as described previously [16]. The eluted peptides were dried under vacuum and stored at −80°C until mass spectrometry analysis.

### Mass spectrometry data acquisition

The peptides analyzed on an Orbitrap Fusion Lumos mass spectrometer coupled with EASY-nLC 1200 nano-flow liquid chromatography system (Thermo Fisher Scientific) as described previously [16]. Briefly, peptides of each fraction were reconstituted in 60 µl of 0.5% formic acid and 18 µl of sample was injected for each of three technical replicate runs. Peptides were loaded on a trap column (Acclaim PepMap 100, 100 µm × 2 cm, Thermo Fisher Scientific) and separated on an analytical column (PepMap 2 µm C_18_, 75 µm × 50 cm, Thermo Fisher Scientific). Peptides were separated over 150 min at flow rate of 250 nl/min. Eluted peptides were ionized at a voltage of 2.2 kV. Mass spectrometry data was obtained in a data-dependent acquisition method with 3 s cycle time. MS scans were measured *m/z* range of 300–1500 at resolution of 60 000. Precursor ions were isolated with 1.6 *m/z* and fragmented with higher-energy collisional dissociation setting of 32. Tandem mass spectrometry (MS/MS) scans were acquired with resolution of 30 000. HeLa protein digest (Thermo Fisher Scientific, 88328) was analyzed between cell types for quality control of chromatography and mass spectrometry.

### Mass spectrometry data analysis

All raw mass spectrometry data were loaded on MaxQuant suite (version 1.6.5.0) and searched against neXtProt database (released in January 2018) with Andromeda search engine. Enzyme specificity was set to trypsin with maximum of two missed cleavages. Carbamidomethylation on cysteine was set as a fixed modification, and N-terminal acetylation, oxidation on methionine, phosphorylation of serine, threonine, and tyrosine were set as variable modifications. FDR 1% was applied for both peptide and proteins using a target-decoy approach. Normalized label-free quantitation (LFQ) values generated by the MaxLFQ algorithm were used for quantitative analysis, and the average LFQ values from three replicates was used for each protein. No bath effect correction was performed. Bolt search engine (version 0.99) was conducted using Pinnacle interface (Optys Tech Corporation) following the setting described in the original article considering modifications [27]. Trypsin specific cleavage was allowed with 10 ppm for precursor ion tolerance and 20 ppm for fragment ion tolerance. The FDR for modified peptides was controlled identically for peptides without modifications with 1% FDR using Percolator. To further increase the confidence of post-translational modification (PTM) identifications, we manually inspected MS/MS spectra of modified peptides and included an additional filtering step. Peptides were required to have annotated fragment ions covering at least 50% of the amino acid sequence (number of annotated residues divided by total peptide length > 0.5). The number of filtered peptides with PTMs are summarized in Supplementary Table S7. We built a custom protein sequence database incorporating novel junction peptides from our RNA-seq, extended sequences representing potential translational initiation sites and translational readthrough events. Alternate canonical and non-canonical translation initiation sites (TIS) of annotated proteins was constructed as described in our previous study [28]. Micro ORFs in 5′-UTR and translational readthrough regions at the stop codon were built using the GENCODE annotations. Mass spectrometry data was searched against the cell type specific database using Bolt search engine. MS/MS scans that were not assigned to any peptide but were identified by Bolt were used for PTM analysis.

### Peptide synthesis

The peptides were synthesized using standard FMOC chemistry on a MultiPep RSi (CEM Corp. Matthews, NC) multiple peptide synthesizer at the 0.025 mmol scale. The starting resin for the light peptides were FMOC-Arg(pbf)-Wang resin or FMOC-Lys(Boc)-Wang resin (Novabiochem). For peptides with PTMs, the following derivatives were used: Fmoc-Lys(Ac)-OH (CreoSalus) for lysine acetylation, Fmoc-Ser(Ac)-OH for serine acetylation, Fmoc-His(Me)-OH for histidine methylation, Fmoc-Lys(Me, Boc)-OH, Fmoc-Lys(Me)_2_-OH, Fmoc-Lys(Me_3_Cl)-OH (Sigma–Aldrich), for lysine methylation, dimethylation, and trimethylation. The peptides were cleaved using the Razor cleaving apparatus (CEM Corp). Cleavage cocktail was trifluoroacetic acid, water, triisopropylsilane, and 3,6-dioxa-1,8-octanedithiol (92.5/2.5/2.5/2.5 v/v/v/v). Peptides were precipitated and washed in cold methyl tert-butyl ether. Each peptide was purified using high-performance liquid chromatography, and its molecular weight was verified with mass spectrometry.

### Immunohistochemical labeling

Validation of the protein encoded by C1orf116 was performed using tissue microarrays (TMA). The slides were baked overnight at 65°C prior to deparaffinization. The tissue sections were deparaffinized in xylene (2 × 10 min) followed by absolute alcohol (5 min) and 95% alcohol (5 min). Following this, the sections were transferred to a 3% v/v solution of hydrogen peroxide in methanol for 20 min for blocking endogenous peroxidases. The sections were then transferred to 70% alcohol (2 min) followed by 0.05 M Tris-buffered saline (TBS), pH 7.6. Antigen retrieval was carried out using citrate buffer (10 mM citric acid, 0.05% Tween 20, pH 6.0) in a pressure cooker for 20 min. The slides were allowed to cool down to room temperature. The slides were transferred to TBS. A solution of 2.5% normal horse serum was applied to the tissue sections for 30 min to block endogenous biotin. The primary antibody anti-C1orf116 (PA5-52867, Thermo Fisher Scientific) was applied to the slides at a dilution of 1:100 and incubated for 2 h at room temperature. The slides were washed in TBS (two changes, 5 min each). A horseradish peroxidase-conjugated polyclonal IgG antibody was used as the secondary antibody (Vector Laboratories, Inc., Burlingame, CA, USA). The slides were incubated with the secondary antibody for 30 min and then washed in TBS (two changes, 5 min each). The slides were then incubated for 5 min in a 1% solution of 3,3′-diaminobenzidine peroxidase substrate. The slides were washed in distilled water (two changes, 2 min each), counterstained with Harris hematoxylin for 30 s, and washed in running tap water for 2 min. Dehydration and clearing were done by incubating the slides in sequential order in 95% alcohol for 2 min and absolute alcohol (2 changes for 3 min each). Clearing was performed in xylene (two changes, 5 min each). Sections were mounted using DPX and appropriate coverslips and incubated at 50°C for 15 min for drying. The slides were examined by a pathologist for the intensity and distribution of staining in the tissue cores in all the TMA.

## Results and discussion

### Generation of transcriptomic and proteomic data from primary human cell types

**Figure 1.**
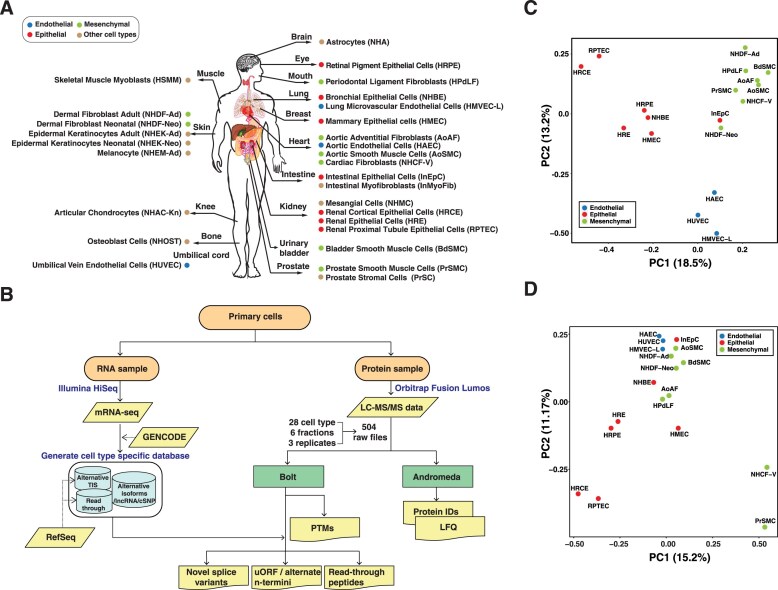
Generation of transcriptome and proteome profiles of primary cells. **(A)** The 28 types of primary cells analyzed in this study. The abbreviation for cell type is noted in parentheses. **(B)** Overall workflow for the transcriptomic and proteomic data generation and analysis. **(C)** Principal component analysis (PCA) plot of mRNA expression for different cell types. **(D)** PCA plot of protein expression for different cell types. Subsets of the 28 cell types were used for the analysis.

We profiled 28 histologically normal cultured human primary cell types using RNA sequencing and mass spectrometry-based label-free quantitative proteomics. Diverse cell types encompassing multiple tissues were selected including seven epithelial cell types, three endothelial cell types, five fibroblast cell type, three smooth muscle cell types, two keratinocyte cell types, and one cell type each of myoblasts, myofibroblasts, melanocytes, chondrocytes, astrocytes, mesangial, osteoblasts, and stromal cells (Fig. 1A and Supplementary Table S1). An overall workflow for the transcriptomic and proteomic analysis of cell types is described in Fig. 1B. Transcriptome analysis, with ∼129 million reads per cell type, resulted in detection of a total of 20 414 protein-coding genes with an average of 16 956 genes per cell type (Supplementary Table S2). Mass spectrometry-based proteomics analysis yielded a total of ∼33 million MS/MS scans from 504 liquid chromatography tandem mass spectrometry (LC-MS/MS) runs, resulting in the identification of 10 480 proteins (an average of 5436 proteins per cell type) mapped to 9698 protein coding genes (Supplementary Fig. S1 and Supplementary Table S3). Among them, 9326 protein coding genes were expressed in both the transcriptome and proteome data.

Principal component analysis (PCA) revealed that cells are clustered by cell type clusters (i.e. endothelial, epithelial and mesenchymal cell types) rather than tissue types in both transcriptome and proteome data (Fig. 1C and D). Three endothelial cell types (HAEC, HUVEC, and HMVEC-L) clustered together. Most epithelial cell types clustered together except InEpC and mesenchymal cell types again showed closer similarity. Interestingly, despite NHCF-V, AoAF, AoSMC, and HAEC all originating from the heart, HAEC was closely clustered with other endothelial cell types in both transcriptome and proteome data. Other uncommon cell types did not cluster with any of these common cell types, indicating distinct molecular profiles and cell physiology for these cell types (Supplementary Fig. S2A). Further, profiling of primary cells enabled the assessment of cellular heterogeneity in tissue, as demonstrated by analyzing the similarities among four distinct cell types derived from kidney. We observed that similarity between the epithelial cells (HRCE, HRE, and RPTEC) and the mesangial cell (NHMC) is lower than between each pair of epithelial cells in both mRNA and protein expression (Supplementary Fig. S2B). This is because mesangial cells are specialized pericytes or smooth muscle cells that contribute to the capillary structure of the glomerulus along with endothelial cells [29, 30]. Overall, our data provide evidence of heterogeneity within the same tissue.

We next examined the correlation between mRNA and protein expression across samples using 6157 protein-coding genes for which both mRNA and protein expression data were available for at least 25% of cell types. Among these genes, 5378 genes (87%) showed positive correlation and 1769 genes (29%) showed significant positive correlation between mRNA and protein abundance. The average mRNA and protein abundance correlation was 0.37, which is consistent with the previous studies [6, 9, 10]. (Supplementary Fig. S2C). We also evaluated the correlation per sample and again observed an average correlation of 0.44. Interestingly, mesenchymal cells showed a significantly lower correlation than epithelial and endothelial cells, which suggests potential differences in post-transcriptional regulation between cell types (Supplementary Fig. S2D).

### Transcriptome data shows cell type enriched expression of molecules

**Figure 2.**
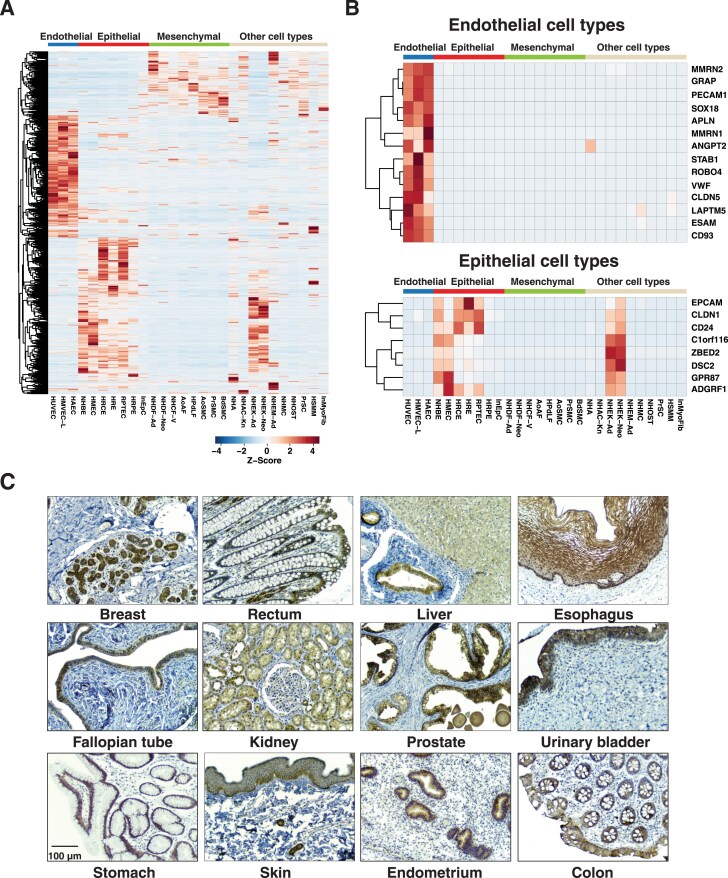
Cell type specific genes demonstrated at mRNA level. **(A)** Heat map of genes with cell type specific expression. **(B)** Heat map of selected genes with endothelial or epithelial cell type specific expression. **(C)** Immunohistochemistry staining of protein C1orf116 across human tissues showing its epithelial cell type specific expression. The panel shows a magnified view (20×) from tissue microarrays.

To identify molecules that are cell type specific, we performed a differential expression analysis of the genes identified at the transcriptome level. Pairwise comparisons of gene expression in each cell type against other cell types were performed, followed by unsupervised clustering of differential genes to identify cell type specific genes. Thousands of genes were found to be overexpressed in a cell type specific manner (Fig. 2A). Overall, we identified 688, 819, and 375 genes specific to endothelial, epithelial, and mesenchymal cell types, respectively (Supplementary Table S4). A gene set enrichment analysis to evaluate the biological processes of endothelial cell type specific genes revealed blood vessel morphogenesis and vascular development as two of the most significantly enriched biological processes (*P*-value <.001). We identified previously reported markers of endothelial cells including PECAM1, CD93, and ESAM [31–33] (Fig. 2B). Similarly, several known epithelial specific molecules were detected including CD24, CLDN1, and EPCAM. Genes with restricted expression in epithelial cell types were involved in biological processes such as cell-cell adhesion and extracellular matrix organization. We found that 180, 364, and 156 cell type–specific molecules identified in our study were also reported in the HPA, which reports 554, 3555, and 900 elevated genes in endothelial, epithelial, and mesenchymal cells, respectively. Notably, 508, 455 and 219 cell type enriched genes that we identified were not explicitly annotated as cell type enriched in HPA. We further examined the trend in proteins encoded by cell type enriched transcripts. Among the 688, 819, and 375 endothelial-, epithelial-, and mesenchymal-enriched genes identified at the mRNA level, 453, 402, and 272 corresponding proteins were detected in the proteomic data, showing similar expression trends for the selected molecules (Supplementary Fig. S3). Similar analyses were conducted to identify genes specific to each tissue type, resulting in the identification of 551 genes specific to kidney, 201 to heart, 102 to prostate, and 369 to skin (Supplementary Fig. S4 and Supplementary Table S5). Notably, among the 551 genes specifically expressed in kidney, 30 solute carrier family genes were detected including SLC12A1, and SLC17A1, which are known to be associated with transporting ions and organic molecules along the renal tubule [34].

One of the epithelial cell type enriched molecules identified from the transcript data is encoded by *C1orf116*, a putative open reading frame that encodes a protein of 601 amino acids. Expression of this protein was originally reported in a human prostate cancer cell line as an unknown cDNA fragment originally designated 21.1 and now referred to as *SARG* [35, 36]. We further examined the expression and distribution of C1orf116 protein using a specific antibody directed against C1orf116 in TMA using immunohistochemistry. The epithelium of several tissue types showed weak to moderate cytoplasmic staining. Its expression was restricted to epithelial cells and was observed to be present in epithelial cells of breast, rectum, liver, esophagus, fallopian tube, kidney, prostate, urinary bladder, stomach, skin, endometrium, and colon tissues (Fig. 2C). Overall, our analysis revealed that C1orf116 is specific to normal epithelium and could be used as a novel epithelial-specific marker in the same manner that EpCAM is used in many studies.

In addition, we identified a total of 24 782 known (12 921 GENCODE and 11 861 FANTOM-CAT) and 6841 novel (number of exons > 1; length ≥ 200 bp; Transcripts Per Million (TPM) ≥ 0.1) lncRNA from 28 cell types. Unsupervised analysis of known lncRNAs showed a clear clustering of endothelial, epithelial and mesenchymal cells, which was in agreement with the results of mRNA (Supplementary Fig. S5A). We observed 734 lncRNAs enriched in endothelial cells while 318 were enriched in epithelial cell types (Supplementary Fig. S5B). Unlike the mRNA of protein-coding genes where ∼80% of genes were expressed in all cell types, we observed that only a small subset of 9023 lncRNAs (28%) were expressed ubiquitously. Further investigations are required to understand the regulation of lncRNA expression and its associated functions.

### Identification of missing proteins with proteomic evidence

**Figure 3.**
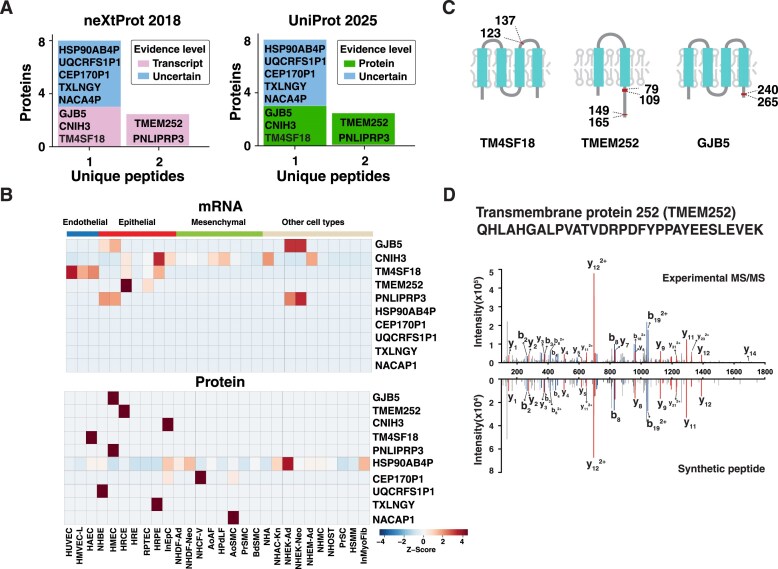
Annotation of missing protein coding genes. **(A)** Bar graph of the number of unique peptides of missing proteins. **(B)** Heat map of missing protein at mRNA and protein level. **(C)** Protein domain structure of protein TM4SF18, TMEM252, and GJB5. Regions of identified peptides are depicted as red boxes. **(D)** The representative experimental MS/MS spectrum of peptide QHLAHGALPVATVDRPDFYPPAYEESLEVEK from protein TMEM252 along with annotated MS/MS spectrum of a synthetic peptide.

Proteome profiling across various primary cell types provided an opportunity to detect missing proteins, which are defined as unconfirmed genetic sequences with evidence at the protein level. Identified proteins from all cell types were mapped to the missing protein list from neXtProt database [37, 38]. After manually inspecting MS/MS spectra of peptides mapped as missing proteins, we observed confident peptide level evidence of 10 proteins. Two proteins (TMEM252, and PNLIPRP3) were identified with two unique peptides, while eight other proteins were identified with one unique peptide (Fig. 3A and Supplementary Table S6). Among these, five proteins (GJB5, CNIH3, TM4SF18, TMEM252, and PNLIPRP3) were classified in the protein existence level 2 (PE2) category, which is based on transcript evidence, while the others were categorized as PE5 indicating uncertain evidence based on the neXtProt protein database we used for the analysis While these molecules were detected in most of cell types at mRNA level, most of them except HSP90AB4P were detected at protein level in only one cell type (Fig. 3B). It indicated the difficulty of detecting peptides because of several reasons. Three proteins (TM4SF18, TMEM252, and GJB5) contain multipass transmembrane domain, which make them difficult to be lysed from the cells because of their amphipathic nature (Fig. 3C). In addition, TMEM252 was shown as an epithelial cell type and kidney specific molecule both at the mRNA and protein level. It indicated that missing proteins could be identified in a few cell types, therefore in-depth proteome profiling of rare cell types increases the chance of detecting such molecules. We further confirmed the identification of proteins using synthetic peptides. A representative example is peptide QHLAHGALPVATVDRPDFYPPAYEESLEVEK of protein TMEM252 showing the same fragment pattern between MS/MS spectrum of experimental and the corresponding synthetic peptide (Fig. 3D). Although we used the strong detergent of 8 M guanidine hydrochloride to improve the lysis efficiency of transmembrane proteins, the limited peptide coverage observed for multipass transmembrane proteins is likely due to the absence of tryptic cleavage sites within their long hydrophobic transmembrane regions. We further validated proteins (NACA4P, GJB5, and TXLNGY) that were identified based on a single unique peptide using synthetic peptides (Supplementary Fig. S6). It should note that during the preparation of this manuscript, several of the above-mentioned proteins (TMEM252, GJB5, TM4SF18, PNLIPRP3, and CNIH3) were reported in a study with protein level evidence in the UniProt database validating our findings (Fig. 3A).

### Landscape of modified peptides in human primary cells

**Figure 4.**
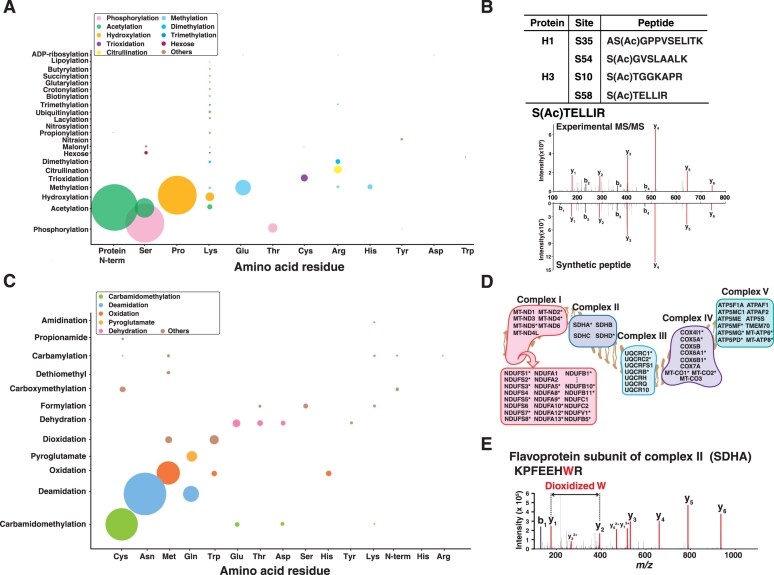
A landscape of modified peptides. **(A)** Frequency of PTMs per residues. Size of circles represents number of modified sites. **(B)** Identified O-acetylated serine sites of histone proteins. The annotated MS/MS spectrum of peptide STELLIR of H3 S58 acetylation is shown along with MS/MS spectrum of a synthetic peptide. **(C)** A bubble chart showing frequency of post-isolation modifications across residues. **(D)** Mitochondrial respiratory chain complexes showing representative proteins. Proteins identified with peptides containing dioxidized tryptophan are indicated with an asterisk. **(E)** An annotated MS/MS spectrum of KPFEEHWR with dioxidized tryptophan from flavoprotein subunit of complex II.

In addition to measuring protein abundance, characterizing various PTMs is required to understand cell biological processes especially signaling pathways, which cannot be extrapolated from the transcriptome [39]. Thus, mass spectrometry data were searched using the Bolt search engine considering 39 types of modifications, which is generally not supported by other search engines because of limitation in computational resources [27, 40]. We classified these 39 modifications into 27 types of biologically relevant PTMs and 12 types of post-isolation modifications that can occur during sample preparation (Supplementary Fig. S7). This resulted in the identification of a total of modified peptides comprising 12 891 peptides with PTMs and 65 148 with post-isolation modifications, which is the largest catalog of modifications of primary cells (Supplementary Table S7). First, we evaluated the overall frequency of identified PTMs at the modified site level (Fig. 4A). As expected, protein N-terminal acetylation and phosphorylation on serine were the most abundant PTMs that we detected. The third most frequent modification was hydroxylation on proline which closely agrees with a previous study that performed PTM search of draft map of human proteome dataset using TagGraph [41] (Supplementary Fig. S8).

Our approach of considering multiple PTMs offered an opportunity to study less investigated PTMs such as acetylation of serine and methylation on glutamic acid and histidine. O-acetylation on serine or threonine residues was first reported in the study of *Yersinia* bacteria [42], and several sites on human histone have been reported [43]. In particular, the acetylation on serine 10 of histone H3 has been shown to be involved in modulating biological functions related to cell cycle progression. We observed the same acetylated site of peptide STGGKAPR from aortic endothelial cells. Further, we detected six O-acetylated peptides of histone proteins, among them four peptides were confident identifications validated with synthetic peptides of histone H1 and H3 (Fig. 4B and Supplementary Fig. S9). To the best of our knowledge, this is the first report of O-acetylation at S57 of H3 and at S36 and S55 of H1 extending the previous observation of H3 S10 O-acetylation.

Although histidine methylation on myosin was first discovered in early 1970s [44], methyltransferase on histidine and their unexpected frequent occurrence have only been reported very recently [45]. We detected a total of 257 peptides with histidine methylation at 266 sites of 230 proteins. Compared to previously reported sites, we identified 229 unreported sites in addition to reconfirming 37 sites including ACTB H73 (Supplementary Fig. S10A and B). We validated a set of histidine methylation sites using synthetic peptides, which confirmed confident matches of fragment ions including detection of immonium ion of methylated histidine residues (i.e. 124.09 m/z).

Next, we evaluated the frequency of 12 post-isolation modifications and observed that oxidation of methionine was the most frequent, followed by deamidation of asparagine, as expected (Fig. 4C). Interestingly, a high number of dioxidation on tryptophan (i.e. 3942 peptides) was detected compared to dioxidation on methionine (i.e. 1018 peptides). This observation suggests that dioxidation on tryptophan may have biological relevance rather than being solely a chemical artifact, which prompted us to investigate this further. We found one study that reported 51 peptides containing dioxidized tryptophan from mitochondria from the human heart [46], of which 45 peptides were present in our dataset. The likelihood of oxidative modification of tryptophan is low, as oxidation has been reported to occur as an artifact during sodium dodecyl sulphate–polyacrylamide gel electrophoresis rather than during in-solution digestion [47]. This supports our observation of proteins with oxidized tryptophan are by-products of cellular processes such as oxidative phosphorylation in mitochondria. To test this, we mapped proteins with dioxidized tryptophan identified in our study to 97 proteins comprising complex I–V subunits. This resulted in an overlap of 36 proteins of complex subunits that were found in our data with oxidized form (Fig. 4D and E). Although further investigations will be required, our study indicates that it is necessary to routinely consider oxidation of tryptophan in addition to methionine to identify biologically relevant modifications.

**Figure 5.**
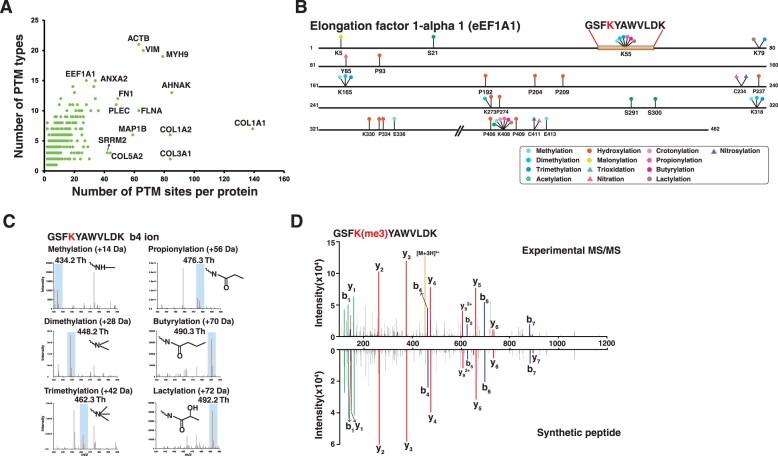
Representative examples of protein with multiple modified sites with various types of PTMs. **(A)** Distribution of the number of sites and the number of PTM types. **(B)** Protein EEF1A1 with 13 types of PTM on 26 sites. **(C)** Peptide level evidence of methylation, dimethylation, trimethylation, propionylation, butyrylation, and lactylation on K55 of EEF1A1 (GSFKYAWVLDK) with their different fragment b_4_ ion mass. **(D)** An annotated MS/MS spectrum of K55 trimethylated peptide of EEF1A along with MS/MS spectrum of a synthetic peptide.

Peptides with PTMs corresponded to 5136 proteins, of which 3059 proteins (60%) have a single modification site. The remaining 2077 proteins were found to have at least two PTM sites (Fig. 5A). Proteins with multiple modified sites primarily consisted of only a few types of PTMs. For example, proteins of the collagen family (e.g. COL1A1, COL3A1, COL5A2, and COL1A2) showed the highest number of modification sites with a significant prevalence of hydroxylation on proline residues, which aligns with previous studies [48–50]. On the other hand, several proteins such as actin and vimentin were shown to have multiple types of PTMs including methylation, acetylation, and hydroxylation. Intriguingly, 13 different types of PTMs on 26 sites were detected in a single protein, eukaryotic translation elongation factor 1 alpha 1 (EEF1A1) (Fig. 5B). Among these, hydroxylation of proline was most frequently observed at 10 sites. Our data reconfirmed methylation of K55, K79, K165, and K318 that were described across several studies of EEF1A1[51–53]. Interestingly, there were several sites such as K55 and K408, which were observed to be modified with multiple types of PTMs including methylation, propionylation, butyrylation, and lactylation. As an illustration, these modifications of K55 were clearly supported by mass difference of b_4_ fragment ion of each modified peptide as shown in Fig. 5C. We further validated them by comparing the fragmentation patterns acquired from synthetic peptides bearing these PTMs (Fig. 5D and Supplementary Fig. S10C). Collectively, we demonstrated that the database search strategy considering various PTMs substantially enriches the landscape of PTMs that are identified. Although we did not observe significant distinction among cell types based on PTMs, mainly because no enrichment strategy was used, we anticipate that such an approaches in routine analyses of global proteomics experiments could eventually benefit the entire proteomics and biochemical research community as advances in mass spectrometry continue to evolve.

### Identification of novel protein-coding regions with integrative analysis of transcriptomic and proteomic data

The use of sample-specific protein database generated from RNA sequencing provides an opportunity for identification of novel protein-coding regions or variants that were missed through conventional protein database searching against reference proteins [16, 54, 55]. We also considered hypothetical sequences of alternative TIS, upstream small open reading frames (uORFs), translational readthrough extensions and splice junctions. This strategy enabled us to identify peptides originating from unannotated regions and noncanonical forms as described below.

### Identification of non-canonical TIS

**Figure 6.**
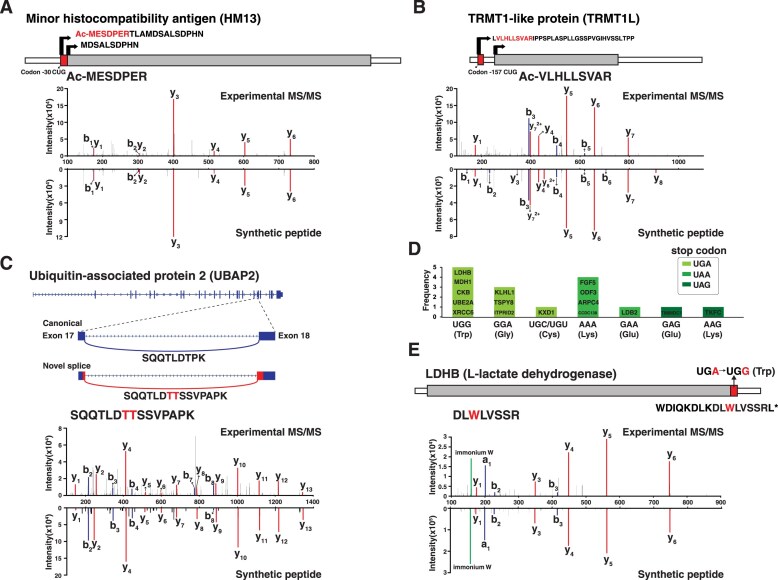
Annotation of novel protein coding regions using proteogenomic analysis. Representative examples of each case are shown with their MS/MS spectra and MS/MS spectra of synthetic peptides. **(A)** HM13 protein identified with upstream alternate N-terminus in-frame with annotated start site (bent arrow) and acetylated peptide MESDPER. **(B)** N-terminal acetylated peptide VLHLLSVAR of upstream of TIS of protein TRMT1L. **(C)** The novel splice junction identified with peptide SQQTLDTTSSVPAPK. **(D)** Bar grpah describing the frequency of stop codon of 16 translational readthrough peptides. **(E)** Translational readthrough of LDHB translating peptide DLWLVSSR by changing stop codon UGA to UGG.

Cells express diverse alternative translation start sites. We have previously demonstrated the possibility of detecting multiple alternate protein N-termini of protein-coding genes, some of which are cell-/tissue-specific [16, 28]. While the canonical start codon AUG codes for methionine, there is increasing evidence of novel TIS originating from non-canonical codons such as ACG, CUG, GUG, or UUG in the 5′ untranslated regions of mRNAs [28]. Ribo-seq based identification of such novel TIS could not reveal if such non-AUG codons are actually translated into corresponding amino acid or methionine. Our recent work demonstrated incorporation of methionine even in the case of non-AUG codons, thereby leading to N-terminal extension of several annotated proteins and led to identification of many translated uORFs. To identify the novel TIS of annotated protein-coding genes and translated uORFs, we generated a putative canonical and non-canonical protein N-termini from three-frame translation of 5′ UTRs as described previously [28]. We identified 11 acetylated peptides from the extensions of annotated N-termini into 5′-UTR, which are indicative of an alternative noncanonical N-termini. For example, TIS was observed for protein minor histocompatibility antigen (HM13) with N-terminally acetylated peptide MESDPER (Fig. 6A). In addition to N-termini extended peptides, 34 peptides originated from uORFs in the 5′ UTRs were detected. Ten peptides were identified from two or more cell types and the remaining 24 were identified only from one cell type. As an example, a novel N-terminally acetylated peptide VLHLLSVAR was identified upstream of annotated TIS of protein TRMT1-like protein (TRMT1L) (Fig. 6B).

### Identification of splice variants

Alternative splicing is a key post-transcriptional regulatory mechanism to generate transcriptome and proteome diversity. It has been reported that alternative splicing contributes to protein complexity in cells to achieve functional diversity and regulates tissue specific protein expression [56]. Here, we sought to identify cell type specific isoforms and peptides with novel splice junction. Several annotated and novel splice junctions were identified. By carrying out analysis of cell type specific splice junctions some interesting patterns of isoform expression were identified. A total of 15 novel splice junctions were identified along with peptide level evidence. In another case, a novel isoform of ubiquitin-associated protein 2 (UBAP2) was identified with a 5′ terminal extension of exon-18 in aortic smooth muscle cell (AoSMC). This splice variant of UBAP2 was identified at mRNA level and additionally supported by proteomic evidence of a novel peptide SQQTLDTTSSVPAPK (Fig. 6C).

### Identification of translational readthrough events using a protein database that includes C-terminally extended peptides.

Decoding stop codons (UAG, UAA, and UGA) as sense codons is known as translational readthrough, which generates C-terminally extended proteins [57]. Especially, programmed translational readthrough that leads to biological function different from the original proteins is termed as functional translational readthrough [58]. We generated a protein database consisting of C-terminally extended peptide sequences by converting the stop codons into 20 amino acids and translating the sequence until the next stop codon to detect peptides resulting from translational readthrough. This approach resulted in the identification of 110 peptides with reliable MS/MS spectra after manual inspection. To prioritize biologically plausible events, we focused on 16 peptides that could arise from a single nucleotide change in the stop codon, excluding those requiring multiple nucleotide substitutions, which are unlikely to occur (Fig. 6D). The most frequent single nucleotide change occurred at UGA followed by UAA and UAG. Notably, we identified several translational readthrough peptides in multiple cell types on proteins such as LDHB and MDH1. For example, we identified the extended peptide DLWLVSSR of LDHB, which was confirmed by the same fragmentation pattern in MS/MS acquired from a synthetic peptide. The stop codon UGA is changed to UGG, which translates into tryptophan and results in the extended peptide sequence detected by mass spectrometry (Fig. 6E). It has been reported that extended proteins of VEGFA, LDHB, and MDH1 generated through functional translational readthrough carry out new functions related to peroxisomal targeting signal, which contribute to redox equivalent regeneration in peroxisomes [58, 59]. This study was performed at genomic level, and to the best of our knowledge, this is the first time demostration of peptide level evidence for these translational readthrough proteins. Clearly, further studies are needed to characterize biological functions of such newly suggested translational readthrough events from our study.

## Conclusions

The human body has cells as the basic units, which are further organized into tissues, organs and organ systems. Despite extensive research, our understanding of the physiology of human tissues and cell types is still not complete. Here, we present the transcriptome and proteome map of 28 primary human cells using RNA-sequencing and high-resolution mass spectrometry. We observed similar features of cell types regardless of anatomical origin. Further, the depth of sequencing allowed us to characterize novel cell type-specific molecules such as an epithelial cell specific molecule, C1orf116. Our strategy of considering various PTMs revealed an enriched landscape of PTMs across cell type including a large number of previously unreported modified sites some of which were further confirmed using synthetic peptides. We should note that a limitation of our study is that although technical replicates were used for proteomic analyses, only single samples were used for each cell type. Thus, larger studies using primary cells from additional individuals might be needed before drawing definitive conclusions, especially for cell type enriched molecules. Nevertheless, we believe that this catalog of transcriptomic and proteomic data will be an invaluable reference for future studies paving the way for a more complete understanding of cell biology.

## Supplementary Material

gkaf1498_Supplemental_Files

## Data Availability

The transcriptome sequencing data have been deposited in NCBI’s Gene Expression Omnibus and are accessible through GEO Series accession number GSE190615 (https://www.ncbi.nlm.nih.gov/geo/query/acc.cgi?acc=GSE190615). The mass spectrometry proteomics data have been deposited to the ProteomeXchange Consortium via the PRIDE partner [60] repository with the dataset identifier PXD062642.
