# Supplementary material for: A transcriptomic and proteomic map of primary human cell types
Source: Nucleic Acids Res. 2026 Jan 14;54(2):gkaf1498. doi: 10.1093/nar/gkaf1498 (PMC12802902; doi:10.1093/nar/gkaf1498)
Supplement: gkaf1498_Supplemental_Files [file gkaf1498_supplemental_files.zip › Supplemenatry Figures.pdf]

SUPPORTING INFORMATION

Supplementary Figure S1. Protein coding genes expressed from (A) transcriptomic and (B) proteomic analysis. On average, 12,969 protein coding genes per cell type were identified using mRNA sequencing and 5,436 protein coding genes were identified in proteomics.

Supplementary Figure S2. (A) PCA considering all 28 cell types. (B) Similarity among four cell types of kidney at transcriptome and proteome level assessed using Pearson correlation. (C) Distribution of Spearman's correlation coefficients of mRNA and protein expression of individual genes across 28 cell types. Gene identified in ≥25% of cell types were used for calculation. Two-sided p-values were calculated to see whether the correlation was significantly different from zero and corrected using the Benjamini–Hochberg false-discovery-rate method was used for calculating p-values. (D) Correlation of mRNA and protein abundance per cell types.

Supplementary Figure S3. Protein expression of cell type-enriched genes. (A) Heat map of selected proteins in showing specific expression in endothelial cell type. (B) Heat map of selected proteins showing specific expression in epithelial cell type. (C) Heat map of selected protein showing specific expression in mesenchymal cell type.

Supplementary Figure S4. Tissue type-enriched genes demonstrated at mRNA level. (A) Heat map of selected genes showing specific expression in kidney. (B) Heat map of selected genes showing specific expression in heart. (C) Heat map of selected genes showing specific expression in prostate (D) Heat map of selected genes showing specific expression in skin.

Supplementary Figure S5. Cell type specific expression of lncRNA. (A) PCA plot of lncRNA showing clusters of cell types. (B) Heat map of overexpressed cell type-specific lncRNA genes.

Supplementary Figure S6. Annotated MS/MS spectra of missing proteins with single peptide hit.

Supplementary Figure S7. Types of post-translational modification and post-isolation modifications considered for Bolt search.

Supplementary Figure S8. A bubble chart showing frequency of post-translational modifications across residues of human proteome map data analyzed with TagGraph.

Supplementary Figure S9. Annotated MS/MS spectra of peptides with serine acetylation. MS/MS spectra of synthetic peptides are shown together.

Supplementary Figure S10. Identification of histidine methylated peptides. (A) Comparison to the previous reported histidine methylated sites. (B) Annotated MS/MS spectra of histidine methylated peptides along with MS/MS spectra of synthetic peptides. (C) Annotated MS/MS spectra of methylated and dimethylated peptides on K55 of protein EEF1A1 along with MS/MS spectra of a synthetic peptide.

Figure S1

A

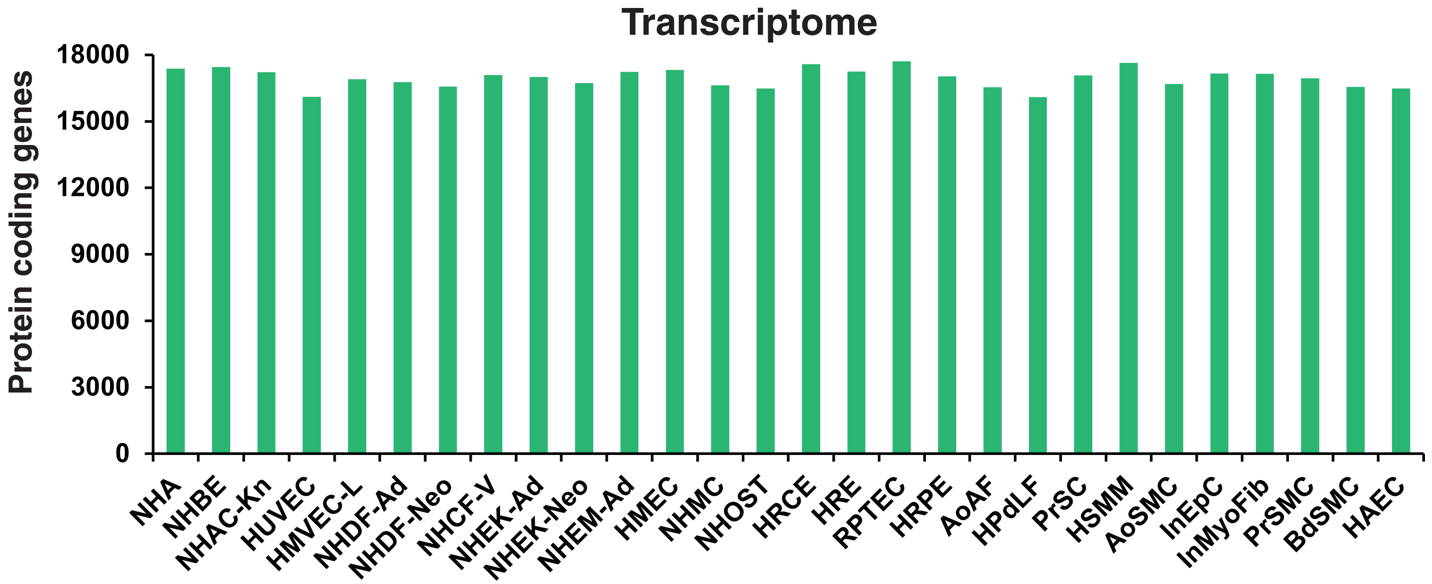
Transcriptome
Protein coding genes
18000
15000
12000
9000
6000
3000
0
NHA
NHBE
NHAC-Kn
HUVEC
HMVEC-L
NHDF-Ad
NHDF-Neo
NHCF-V
NHEK-Ad
NHEK-Neo
NHEM-Ad
HMEC
NHMC
NHOST
HRCE
HRE
RPTEC
HRPE
AoAF
HPdLF
PrSC
HSMM
AoSMC
InEpC
InMyoFib
PrSMC
BdSMC
HAEC

B

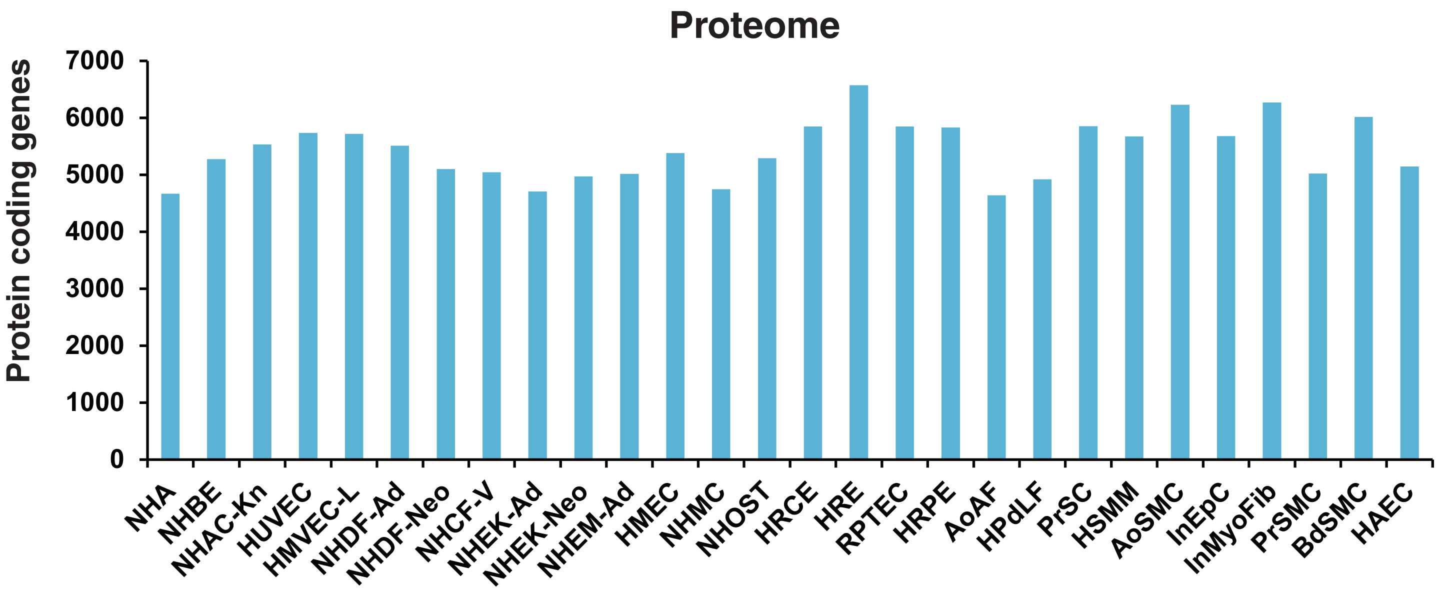
Proteome
Protein coding genes
7000
6000
5000
4000
3000
2000
1000
0
NHA
NHBE
NHAC-Kn
HUVEC
HMVEC-L
NHDF-Ad
NHDF-Neo
NHCF-V
NHEK-Ad
NHEK-Neo
NHEM-Ad
HMEC
NHMC
NHOST
HRCE
HRE
RPTEC
HRPE
AoAF
HPdLF
PrSC
HSMM
AoSMC
InEpC
InMyoFib
PrSMC
BdSMC
HAEC

Figure S2

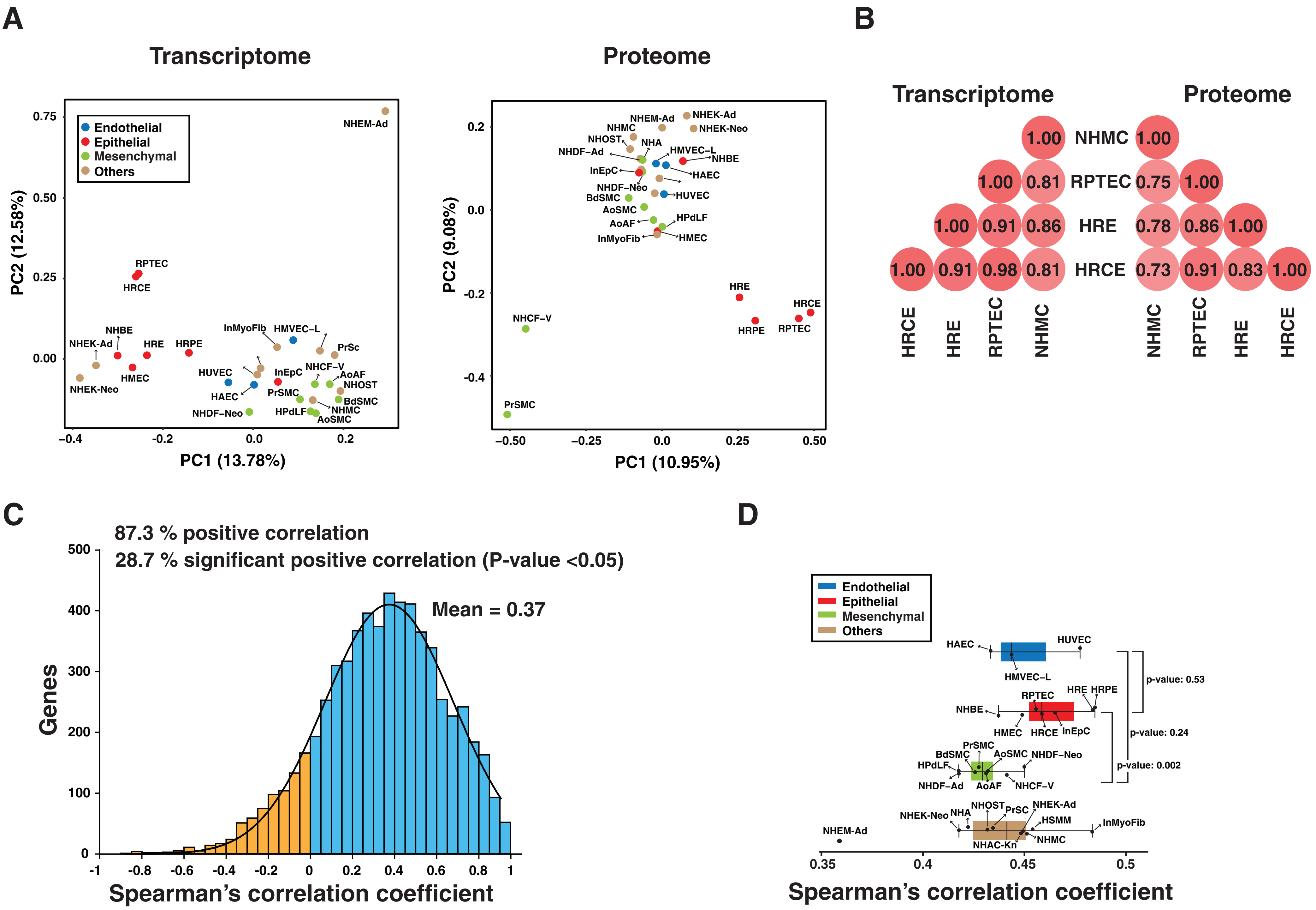

A
Transcriptome
Proteome
Endothelial
Epithelial
Mesenchymal
Others
PC2 (12.58%)
PC1 (13.78%)
PC2 (9.08%)
PC1 (10.95%)
B
Transcriptome
Proteome
NHMC
RPTEC
HRE
HRCE
C
87.3 % positive correlation
28.7 % significant positive correlation (P-value <0.05)
Mean = 0.37
Genes
Spearman's correlation coefficient
D
p-value: 0.53
p-value: 0.24
p-value: 0.002
Spearman's correlation coefficient

Figure S3

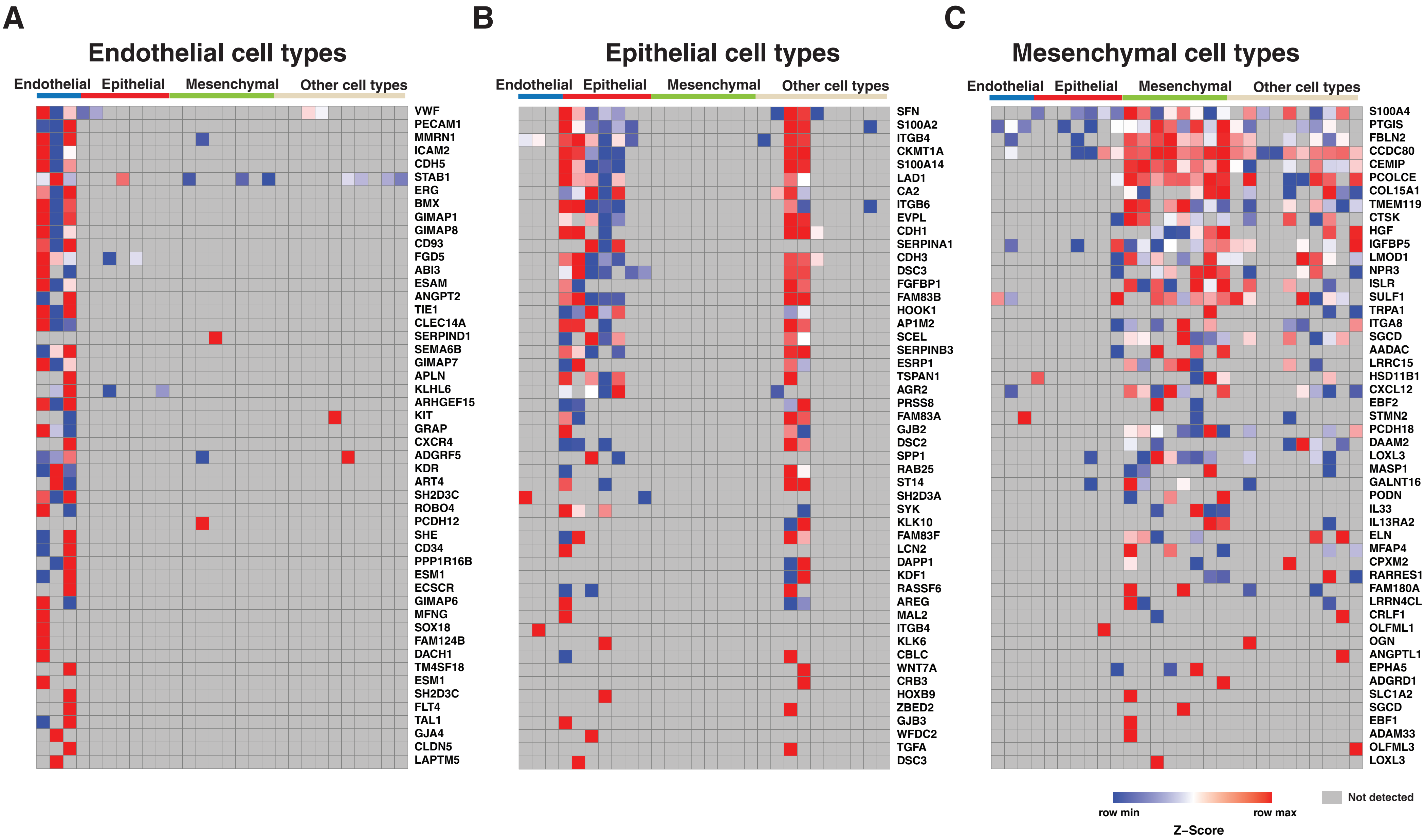
A
Endothelial cell types
Endothelial
Epithelial
Mesenchymal
Other cell types
VWF
PECAM1
MMRN1
ICAM2
CDH5
STAB1
ERG
BMX
GIMAP1
GIMAP8
CD93
FGD5
ABI3
ESAM
ANGPT2
TIE1
CLEC14A
SERPIND1
SEMA6B
GIMAP7
APLN
KLHL6
ARHGEF15
KIT
GRAP
CXCR4
ADGRF5
KDR
ART4
SH2D3C
ROBO4
PCDH12
SHE
CD34
PPP1R16B
ESM1
ECSCR
GIMAP6
MFNG
SOX18
FAM124B
DACH1
TM4SF18
ESM1
SH2D3C
FLT4
TAL1
GJA4
CLDN5
LAPTM5
B
Epithelial cell types
Endothelial
Epithelial
Mesenchymal
Other cell types
SFN
S100A2
ITGB4
CKMT1A
S100A14
LAD1
CA2
ITGB6
EVPL
CDH1
SERPINA1
CDH3
DSC3
FGFBP1
FAM83B
HOOK1
AP1M2
SCEL
SERPINB3
ESRP1
TSPAN1
AGR2
PRSS8
FAM83A
GJB2
DSC2
SPP1
RAB25
ST14
SH2D3A
SYK
KLK10
FAM83F
LCN2
DAPP1
KDF1
RASSF6
AREG
MAL2
ITGB4
KLK6
CBLC
WNT7A
CRB3
HOXB9
ZBED2
GJB3
WFDC2
TGFA
DSC3
C
Mesenchymal cell types
Endothelial
Epithelial
Mesenchymal
Other cell types
S100A4
PTGIS
FBLN2
CCDC80
CEMIP
PCOLCE
COL15A1
TMEM119
CTSK
HGF
IGFBP5
LMOD1
NPR3
ISLR
SULF1
TRPA1
ITGA8
SGCD
AADAC
LRRC15
HSD11B1
CXCL12
EBF2
STMN2
PCDH18
DAAM2
LOXL3
MASP1
GALNT16
PODN
IL33
IL13RA2
ELN
MFAP4
CPXM2
RARRES1
FAM180A
LRRN4CL
CRLF1
OLFML1
OGN
ANGPTL1
EPHA5
ADGRD1
SLC1A2
SGCD
EBF1
ADAM33
OLFML3
LOXL3
row min
row max
Z–Score
Not detected

Figure S4

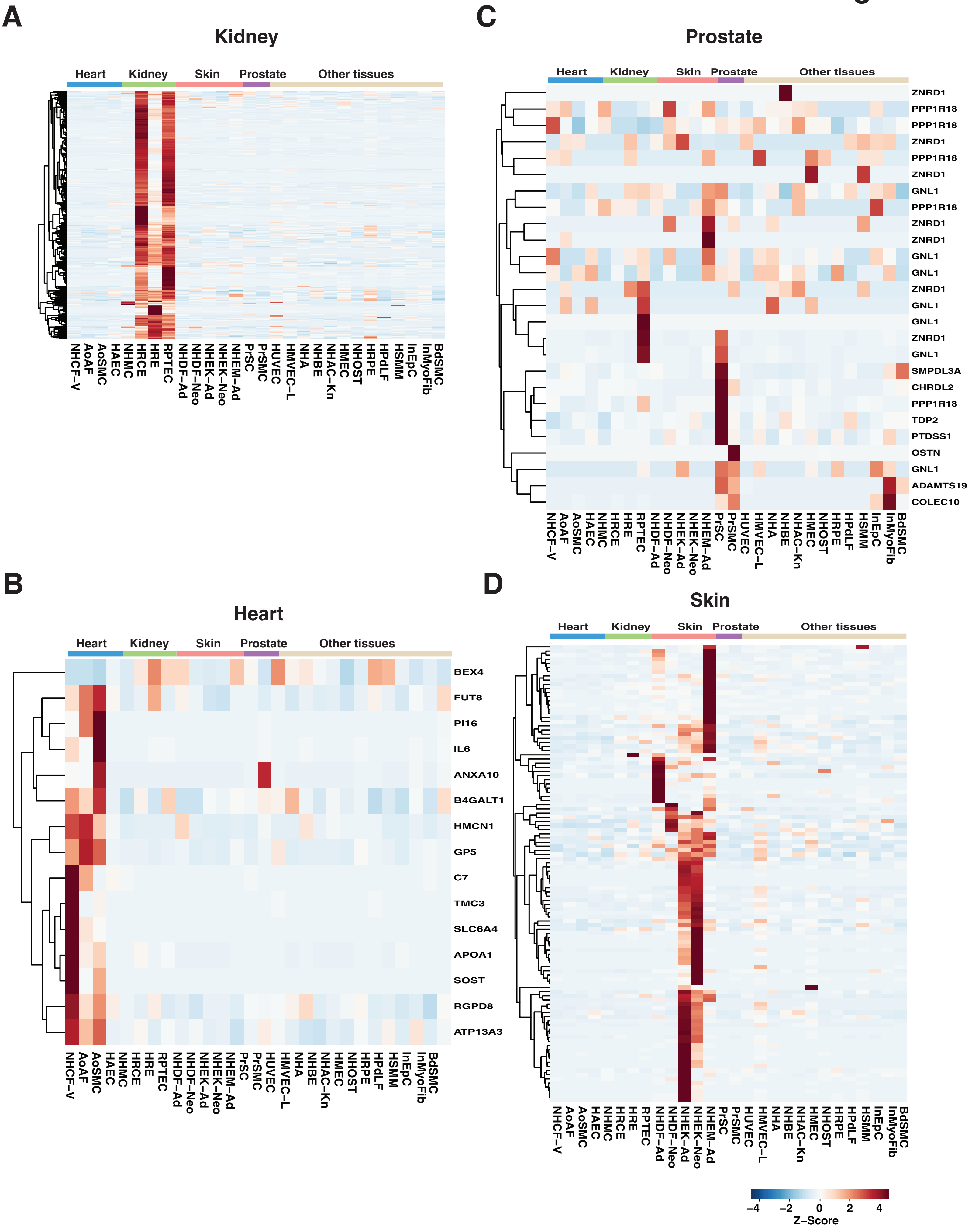
A
Kidney
Heart
Kidney
Skin
Prostate
Other tissues
NHCF–V
AoAF
AoSMC
HAEC
NHMC
HRCE
HRE
RPTEC
NHDF–Ad
NHDF–Neo
NHEK–Ad
NHEK–Neo
NHEM–Ad
PrSC
PrSMC
HUVEC
HMVEC–L
NHA
NHBE
NHAC–Kn
HMEC
NHOST
HRPE
HPdLF
HSMM
InEpC
InMyoFib
BdSMC
B
Heart
BEX4
FUT8
PI16
IL6
ANXA10
B4GALT1
HMCN1
GP5
C7
TMC3
SLC6A4
APOA1
SOST
RGPD8
ATP13A3
C
Prostate
ZNRD1
PPP1R18
PPP1R18
ZNRD1
PPP1R18
ZNRD1
GNL1
PPP1R18
ZNRD1
ZNRD1
GNL1
GNL1
ZNRD1
GNL1
GNL1
ZNRD1
GNL1
SMPDL3A
CHRDL2
PPP1R18
TDP2
PTDSS1
OSTN
GNL1
ADAMTS19
COLEC10
D
Skin
−4
−2
0
2
4
Z–Score

Figure S5

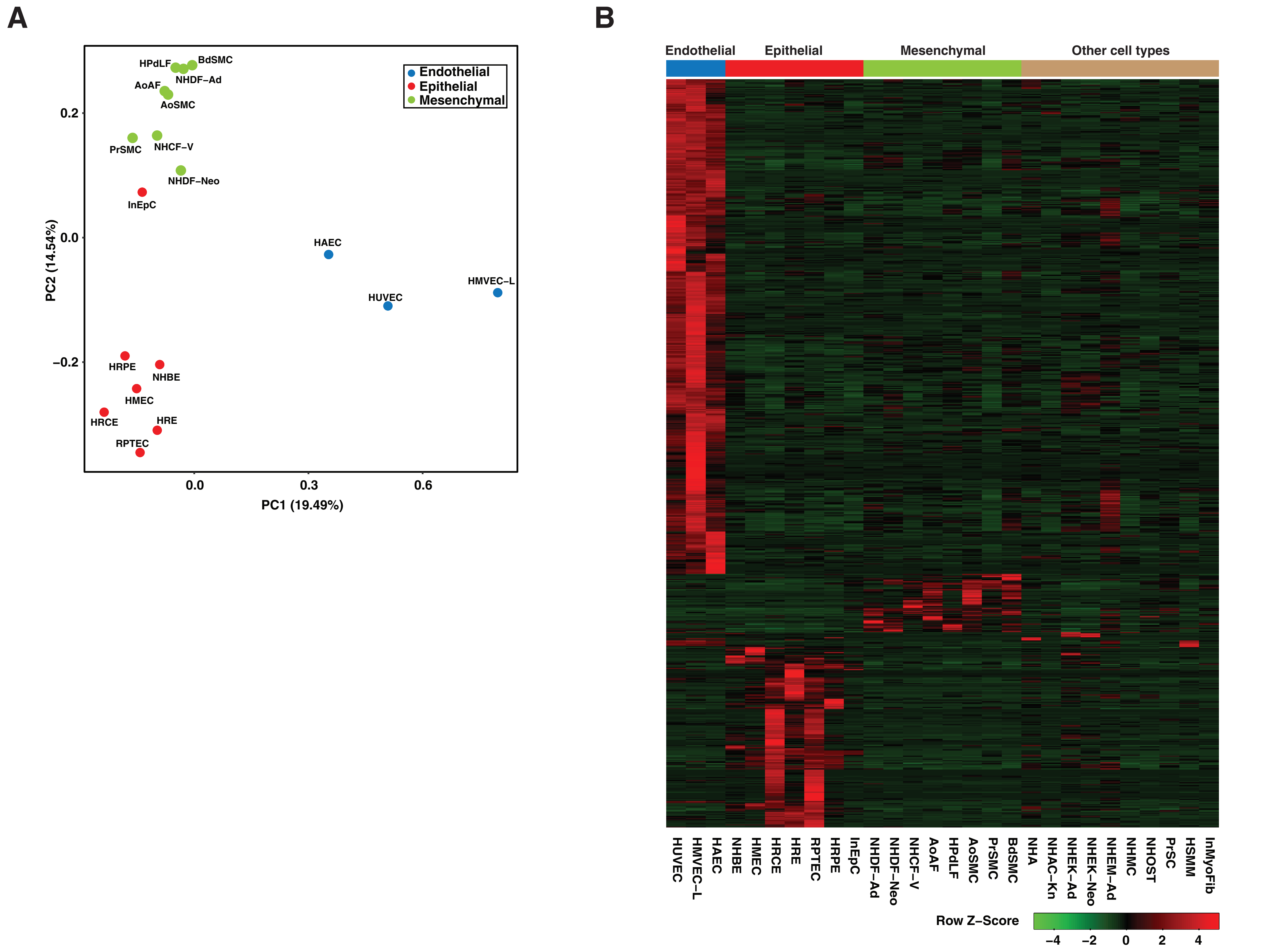

A
Endothelial
Epithelial
Mesenchymal
HPdLF
BdSMC
NHDF–Ad
AoAF
AoSMC
PrSMC
NHCF–V
NHDF–Neo
InEpC
HAEC
HMVEC–L
HUVEC
HRPE
NHBE
HMEC
HRCE
HRE
RPTEC
0.2
0.0
−0.2
0.0
0.3
0.6
PC2 (14.54%)
PC1 (19.49%)
B
Endothelial
Epithelial
Mesenchymal
Other cell types
HUVEC
HMVEC–L
HAEC
NHBE
HMEC
HRCE
HRE
RPTEC
HRPE
InEpC
NHDF–Ad
NHDF–Neo
NHCF–V
AoAF
HPdLF
AoSMC
PrSMC
BdSMC
NHA
NHAC–Kn
NHEK–Ad
NHEK–Neo
NHEM–Ad
NHMC
NHOST
PrSC
HSMM
InMyoFib
Row Z–Score
−4
−2
0
2
4

Figure S6

A

Putative nascent polypeptide-associated complex subunit alpha-like protein (NACA4P)

IEDLSQEAQLAAAEK

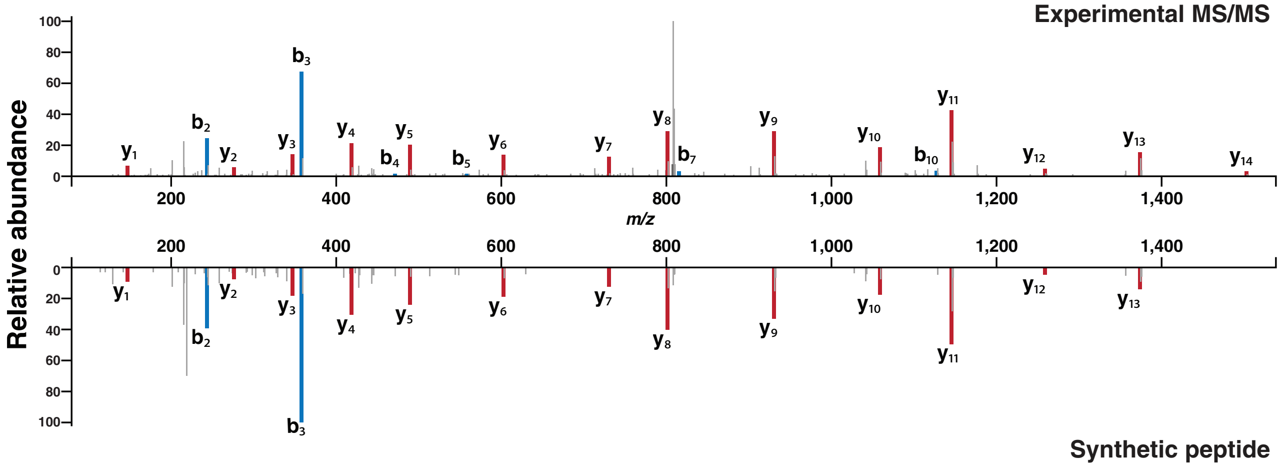

B

Gap junction beta-5 protein (GJB5)

QDDLLSGDLIFLGSDSHPPLLPDRPR

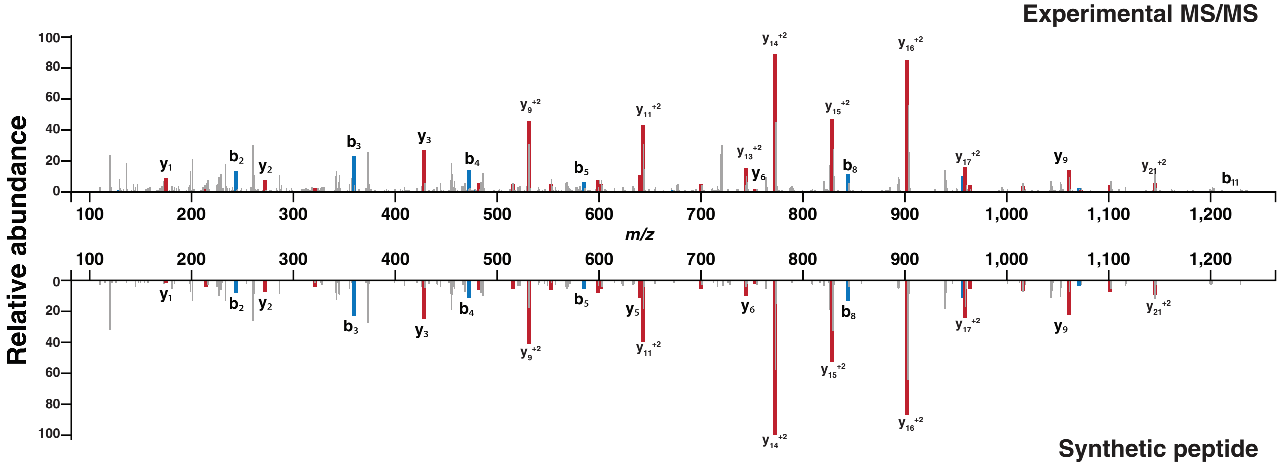

C

Putative gamma-taxilin 2 (TXLNGY)

HLLEDEEGRDFITK

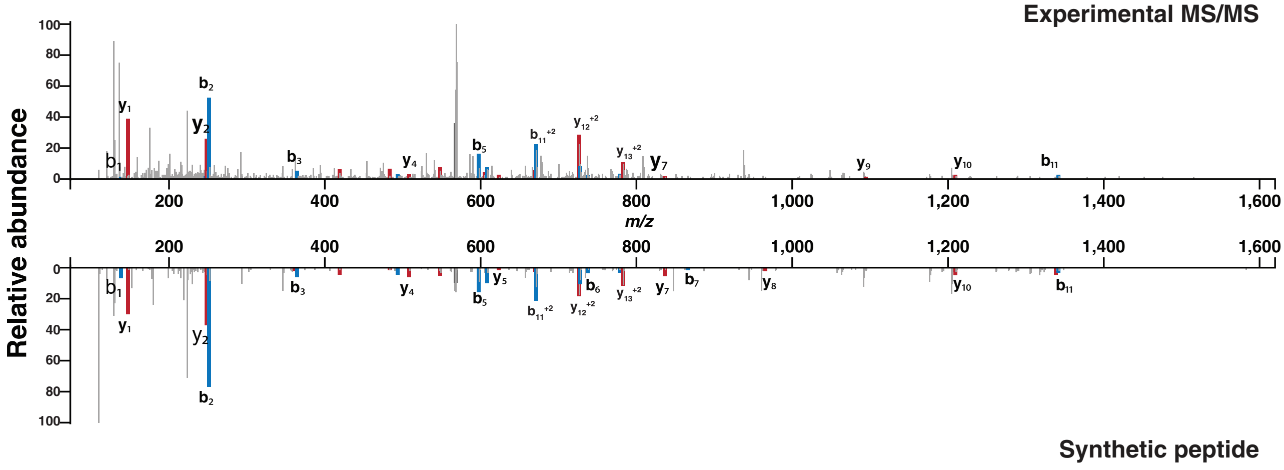

Figure S7

Post-translational modifications

Description	Site
Acetylation	Lys, Ser, Protein N-term, Met-loss N-term
ADP ribosylation	Asp, Arg, Glu
Biontinylation	Lys
Butyrylation	Lys
Citrullination	Arg
Crotonylation	Lys
Farnesylation	Cys
GeranylGeranylation	Cys
Glutarylation	Lys
Hexose	Lys, Ser, Trp
Hydroxylation	Lys, Pro
Lactylation	Lys
Lipoylation	Lys
Malonylation	Cys, Lys, Ser
Methylation	Arg, Glu, His, Lys
Dimethylation	Arg, Lys, N-term
Trimethylation	Arg, Lys
Myristoylation	Protein N-term
Nitrosylation	Cys
Nitration	Tyr
Palmitoylation	Cys
Phosphorylation	Ser, Thr, Tyr
Phosphoate riobosylation	Asp,Arg,Glu
Propionylation	Lys
Succinylation	Lys
Sulfation	Tyr
Trioxidation	Cys
Ubiquitylation (GlyGly)	Lys

Post-isolation modifications

Description	Site
Amidination	Lys
Carbamidomethylation	Asp, Cys, Glu, Lys, N-term
Carbamylation	Arg, Cys, Lys, Met, N-term
Carboxymethylation	Cys, N-term
Deamidation	Asn, Gln
Dehydration	Asp, Glu, Thr, Tyr
Dethiomethylation	Met
Dioxidation	Met, Trp
Formylation	Lys, Ser, Thr
Oxidation	His, Met, Trp
Propionamide	Cys
Pyroglutamate	Glu

Figure S8

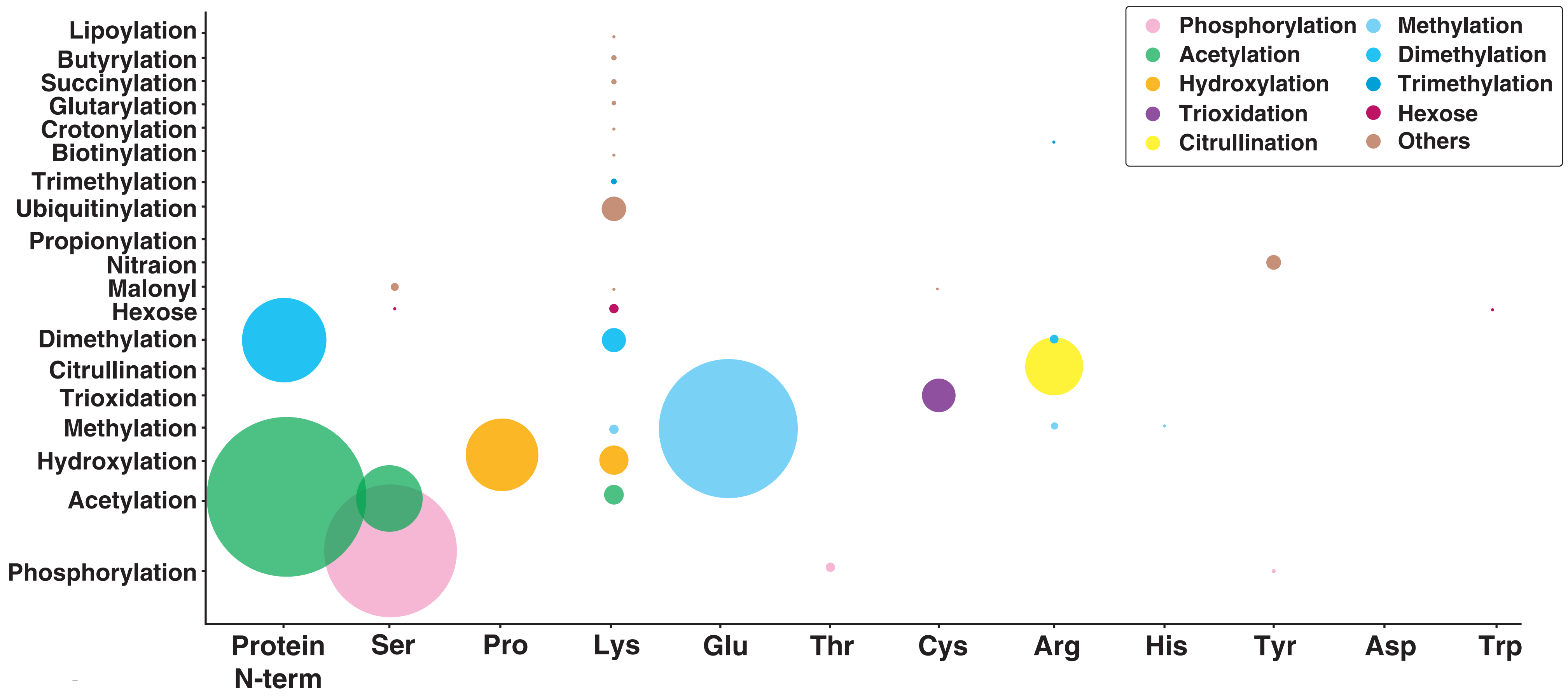

Lipoylation
Butyrylation
Succinylation
Glutarylation
Crotonylation
Biotinylation
Trimethylation
Ubiquitinylation
Propionylation
Nitraion
Malonyl
Hexose
Dimethylation
Citrullination
Trioxidation
Methylation
Hydroxylation
Acetylation
Phosphorylation
Protein N-term
Ser
Pro
Lys
Glu
Thr
Cys
Arg
His
Tyr
Asp
Trp
Phosphorylation
Acetylation
Hydroxylation
Trioxidation
Citrullination
Methylation
Dimethylation
Trimethylation
Hexose
Others

Figure S9

A

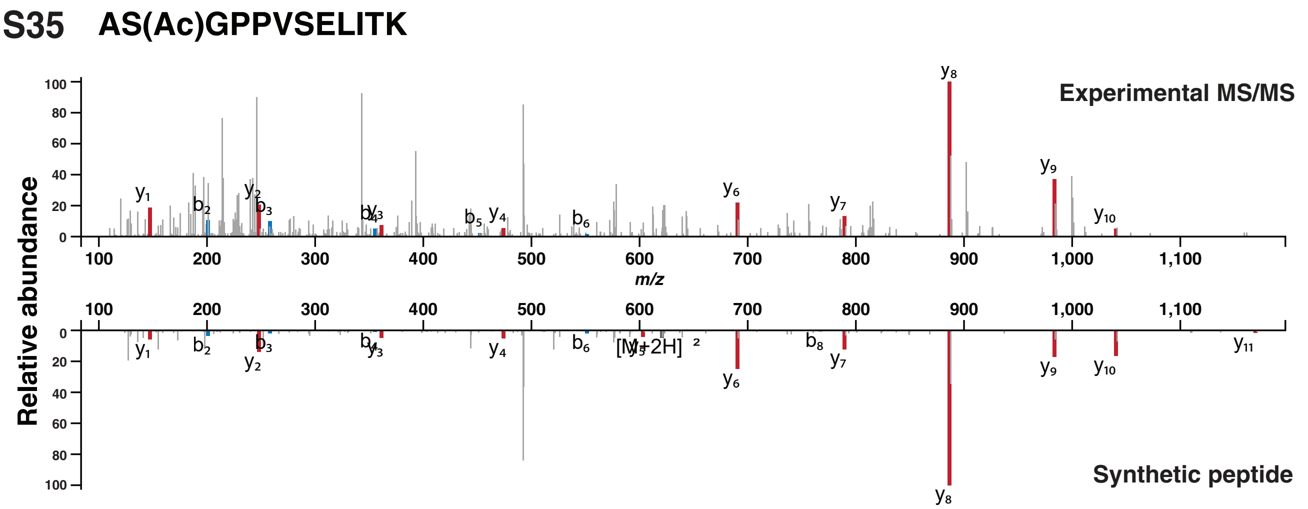

B

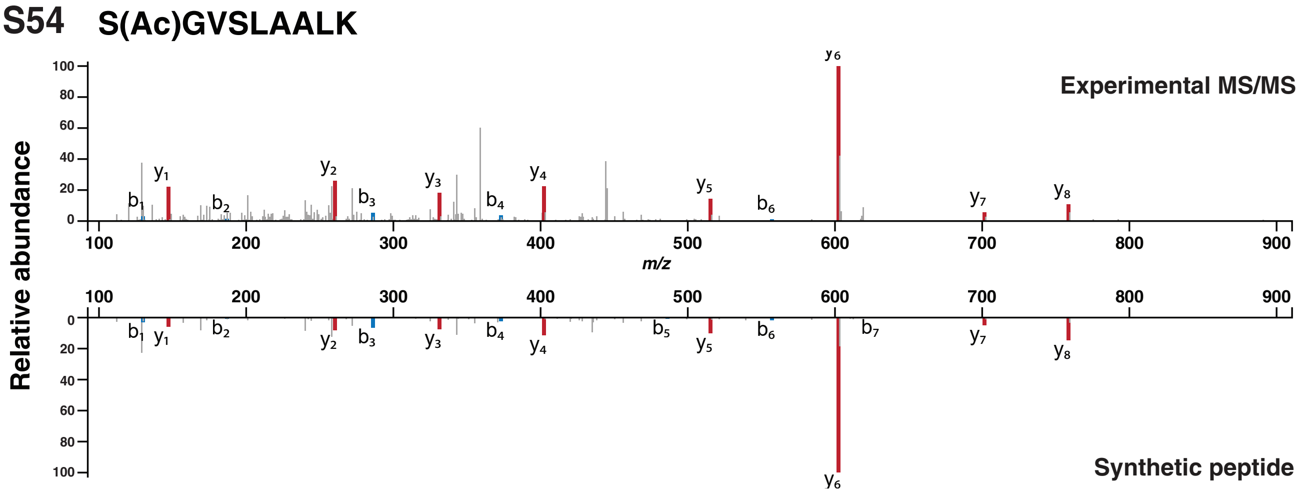

C

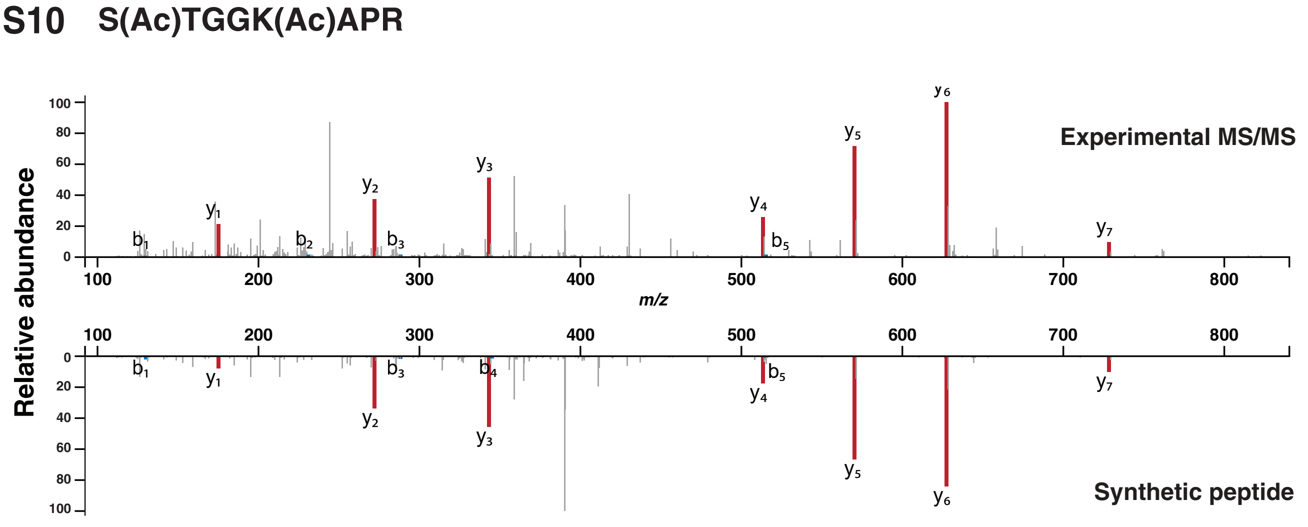

Figure S10

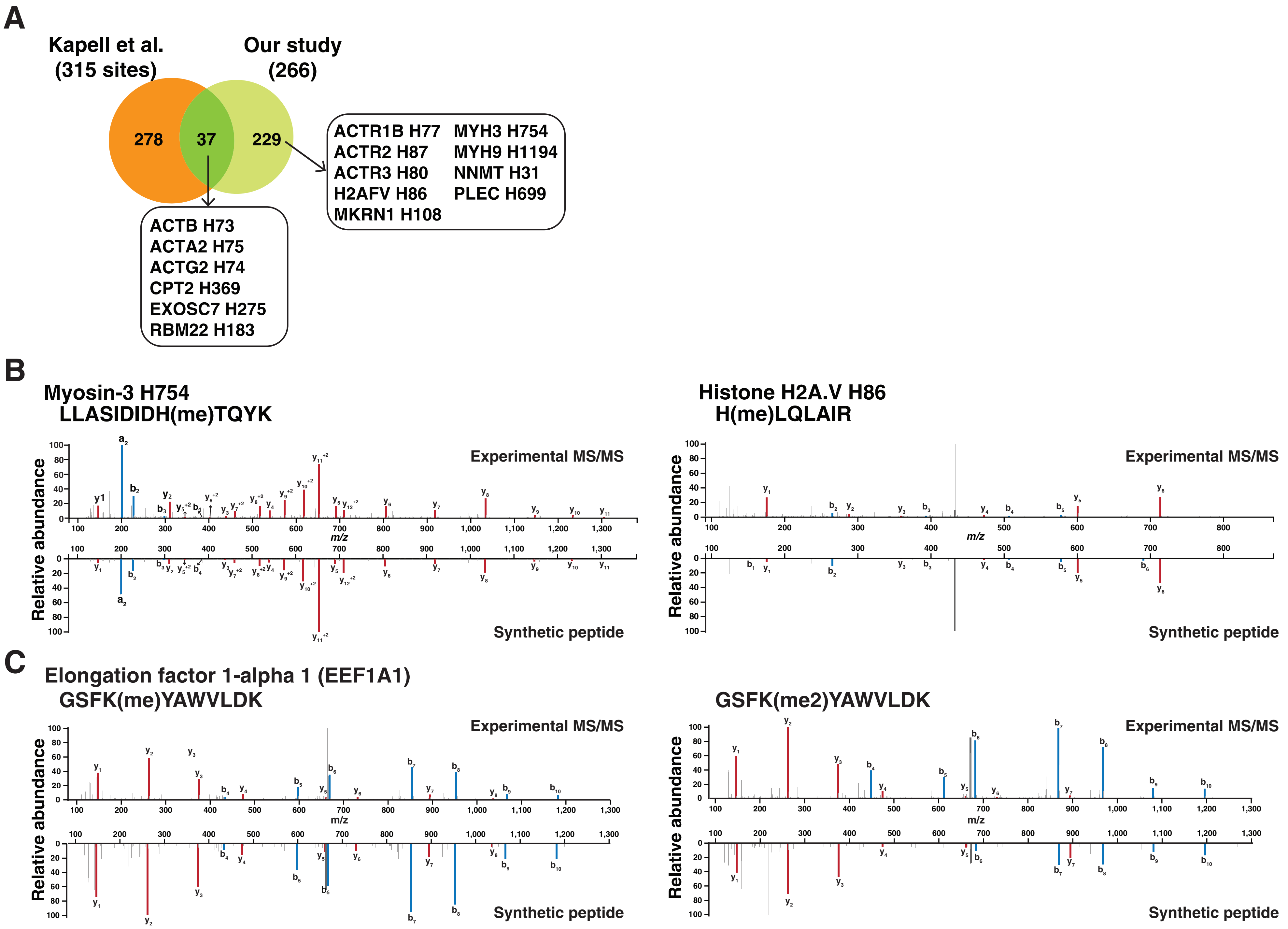
A
Kapell et al.
(315 sites)
Our study
(266)
278
37
229
ACTR1B H77
ACTR2 H87
ACTR3 H80
H2AFV H86
MKRN1 H108
MYH3 H754
MYH9 H1194
NNMT H31
PLEC H699
ACTB H73
ACTA2 H75
ACTG2 H74
CPT2 H369
EXOSC7 H275
RBM22 H183
B
Myosin-3 H754
LLASIDIDH(me)TQYK
Histone H2A.V H86
H(me)LQLAIR
Relative abundance
Experimental MS/MS
Synthetic peptide
m/z
C
Elongation factor 1-alpha 1 (EEF1A1)
GSFK(me)YAWVLDK
GSFK(me2)YAWVLDK
Relative abundance
Experimental MS/MS
Synthetic peptide
m/z
